# The POT-PUFF Sign: A New Angiographic Indicator of Stent Malapposition During Proximal Optimization Therapy

**DOI:** 10.7759/cureus.43552

**Published:** 2023-08-16

**Authors:** Zakariae Laraichi, Raid Faraj, Nadia Fellat

**Affiliations:** 1 Cardiology A Department, Ibn Sina University Hospital/Mohammed V University Rabat, Rabat, MAR; 2 Cardiology B Department, Ibn Sina University Hospital/Mohammed V University Rabat, Rabat, MAR

**Keywords:** pci, coronary angiography, intracoronary imaging, pot-puff sign, stent malapposition

## Abstract

Acute stent malapposition poses a significant risk for adverse cardiac events following percutaneous coronary intervention. Detection of acute stent malapposition traditionally relies on intracoronary imaging techniques, such as intravascular ultrasound and optical coherence tomography, which may be limited in developing countries due to accessibility issues. A new angiographic sign called the *POT-PUFF sign* has been introduced as a potential alternative for detecting malapposition during coronary bifurcation procedures. Here, we present two clinical cases from a developing country where the POT-PUFF sign was employed to assess the result of proximal optimization therapy after stent implantation. The POT-PUFF sign exhibits potential as an affordable and feasible approach for assessing stent malapposition in settings with limited resources.

## Introduction

Acute stent malapposition (ASM) refers to post-procedure lack of contact of stent struts with vessel walls. This condition can be detected using intracoronary imaging techniques such as intravascular ultrasound (IVUS) and optical coherence tomography (OCT). Identifying and addressing malapposition is crucial as it can result in various complications, including impaired stent function, heightened risk of stent thrombosis, and potential adverse cardiac events [1]. Recently, a new angiographic sign called the POT-PUFF sign has been described to detect malapposition in the mother branch during coronary bifurcation procedures based on contrast medium progression through the inflated proximal optimization therapy (POT) balloon to evaluate coronary opacification and flow [2]. Within the context of developing countries, like ours, where access to intracoronary imaging techniques is typically scarce or non-existent, its utility becomes particularly pronounced. In this article, we report two clinical cases wherein the use of this sign enabled us to better assess the result of POT. Our paper was written according to the CARE guidelines [3].

## Case presentation

Case one

**Figure 1:**
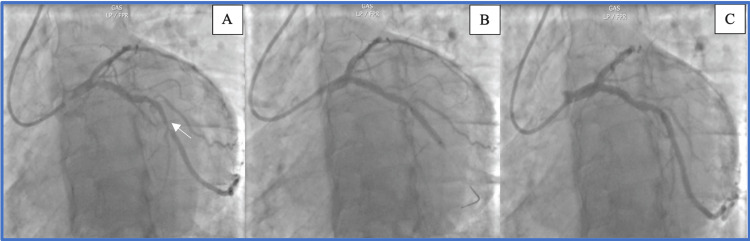
Coronary angiogram findings. A: Severe stenosis of the marginal artery (white arrow). B: Negative POT-PUFF sign as there is no passage of iodinated contrast distally during the four heartbeats following injection. C: Final result.

A 58-year-old man, hypertensive and a weaned smoker, had been suffering from exertional angina for several months. The electrocardiogram showed lateral biphasic T waves. Echocardiography revealed hypokinesia of the anterolateral wall with a moderately impaired ejection fraction of 45%. Coronary angiography revealed significant stenosis at the start of the main marginal artery (Figure 1A). After pre-dilatation of the lesion, tenting was performed using a 3 × 18 mm drug-eluting stent. Post-dilatation was performed using a 3 × 15 mm non-compliant balloon. In the absence of intracoronary imaging in the cardiac catheterization room to assess the correct apposition of the stent, the POT-PUFF sign was performed, showing no passage of iodinated contrast distally during the four heartbeats following injection (Figure 1B). The angiographic result was deemed satisfactory at the end of the procedure (Figure 1C).

Case two

**Figure 2:**
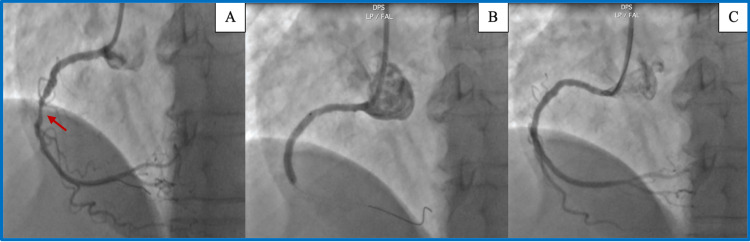
Coronary angiogram findings. A: Severe stenosis of the mid-right coronary artery (red arrow). B: Negative POT-PUFF sign as there is no passage of iodinated contrast distally during the four heartbeats following injection. C: Final result.

Our second case was a 64-year-old hypertensive patient with dyslipidemia on statins. She was admitted for management of chronic coronary syndrome with New York Heart Association class 2 exertional dyspnea. The electrocardiogram was unremarkable. Echocardiography showed left ventricular hypertrophy without segmental contractility abnormalities and a preserved ejection fraction. Stress echocardiography showed ischemia in two segments of the right coronary artery territory. Coronary angiography revealed a long, significant stenosis in the middle segment of the right coronary artery (Figure 2A). After pre-dilatation of the lesion, tenting was performed using a 2.75 × 33 mm drug-eluting stent. Post-dilation was performed using the stent balloon. The POT-PUFF sign was negative (Figure 2B) at the end of the procedure, with an angiographic result deemed satisfactory (Figure 2C).

## Discussion

After stent implantation, IVUS and OCT can identify remediable irregularities associated with both the stent and the underlying vessel wall. These irregularities include stent under-expansion, geographic plaque miss, strut malapposition, and stent edge dissection, and they have been linked to adverse percutaneous coronary intervention (PCI) outcomes [4]. Strut malapposition was characterized as the distance exceeding 200 μm between the strut and the vessel wall, and a distance greater than 400 μm was considered severe strut malapposition. Stent malapposition, on the other hand, was defined as strut malapposition with a length exceeding 1 mm [2]. When faced with ASM, it is crucial to be knowledgeable about the appropriate steps to take. Post-dilatation using a larger balloon or higher pressure is often contemplated by many as a corrective measure for malapposition, particularly when coexisting stent under-expansion is present. Aminfar et al. [2] conducted a successful evaluation of OCT and POT-PUFF signs in 187 non-left-main bifurcations. Its principle relies on the fact that the positive progression of contrast medium (PUFF) through the inflated POT balloon allows for better detection of stent malapposition compared to OCT. Four patients were excluded due to suboptimal OCT quality, which hindered a thorough analysis of the POT segment. Out of the remaining 183 bifurcations, malapposition was observed in 22% of cases when using a 200 μm cut-off, and in 11% of cases when using a 400 μm cut-off. Interestingly, the transition from the positive to the negative POT-PUFF sign resulted in a significant decrease in the risk of malapposition, reducing it from 70.5% to 6.5%. These findings promote the adoption of the POT-PUFF sign as a means to ensure proper stent apposition, particularly in low-income countries where OCT availability is limited, as presented in our cases [2]. The PESTO registry emphasized that strut malapposition is a primary factor contributing to stent thrombosis. This finding underscores the importance of thoroughly evaluating malapposition during bifurcation PCI [5]. Although in our two cases the POT-PUFF sign was not performed in bifurcation lesions, we believe that its use can be extended to several coronary stenting strategies where the use of POT is fundamental to reduce the risk of malapposition. Further studies using endocoronary imaging are needed to confirm or refute this hypothesis.

## Conclusions

The POT-PUFF sign presents a promising new angiographic indicator for detecting stent malapposition during POT after PCI. While further research is required to validate its effectiveness, the POT-PUFF sign shows great potential in enhancing stent apposition evaluation and improving PCI outcomes in resource-constrained settings.
